# Recurrent severe pyogenic infections revealing neutrophil-specific granule deficiency caused by a novel CEBPEsplice-region variant: a case report

**DOI:** 10.3389/fmed.2026.1957544

**Published:** 2026-10-07

**Authors:** Ruxandra Irimia, Onda Calugaru, Ana Maria Forsea, Mara Madalina Mihai

**Affiliations:** 1 Department of Hematology, Emergency Hospital Prof. Dr. Agrippa Ionescu, Bucharest, Romania; 2 Department of Hematology, Institutul Clinic Fundeni, Bucharest, Romania; 3 Department of Dermatology, Spitalul Universitar de Urgenta Elias, Bucharest, Romania; 4 Universitatea de Medicina si Farmacie Carol Davila din Bucuresti, Bucharest, Romania

**Keywords:** CEBA, immundeficiencies, infections, neutrophil, neutrophil granule

## Abstract

Neutrophil-specific granule deficiency is an exceptionally rare autosomal recessive primary immunodeficiency caused by pathogenic variants in the CCAAT/enhancer-binding protein epsilon (CEBPE) gene. Defective neutrophil maturation results in impaired bactericidal activity and recurrent pyogenic infections, predominantly affecting the skin and soft tissues. We describe two affected siblings from a family of four carrying a previously unreported CEBPE missense substitution in the splice region (p.K170N; c.510G>C). A 37-year-old woman presented with lifelong recurrent pyogenic skin infections, including abscesses, ulcerated lesions and extensive atrophic scarring involving multiple body sites. Her brother exhibited a similar phenotype complicated by recurrent osteomyelitis, pulmonary abscesses and permanent osseous deformities. Both patients had experienced recurrent infections since early childhood and had received multiple courses of antibiotics and surgical drainage procedures before the correct diagnosis was established. Peripheral blood smear demonstrated hypogranular neutrophils with bilobed nuclei, while flow cytometry revealed aberrant CD14 expression. Genetic analysis confirmed the novel CEBPE variant in both affected siblings, establishing the diagnosis of neutrophil-specific granule deficiency.

## Introduction

Neutrophil-specific granule deficiency (SGD) is a very rare autosomal recessive immunodeficiency syndrome characterized by abnormal neutrophil morphology and function (1, 2). The condition results from mutations in the CCAAT/enhancer-binding protein epsilon (*CEBPE*) gene, which disrupt the maturation of promyelocytes into myelocytes and of myelocytes into mature neutrophils, thereby impairing the formation of secondary and gelatinase granules (3–9). This structural defect impairs bactericidal function and predisposes affected individuals to recurrent, severe pyogenic infections. The clinical presentation of affected individuals is typically marked by persistent, recurrent cutaneous infections, which may progress to visceral abscesses (10). The lesions are most commonly caused by *Staphylococcus aureus* and *Pseudomonas aeruginosa*, although other enteric Gram-negative bacteria and fungal pathogens such as *Candida albicans* have also been involved (11). Morphologically, the abnormal neutrophils exhibit bilobed nuclei resembling Pelger–Huët anomaly and lack specific granules, while immunophenotypically they display aberrant expression of CD14 and CD64. Genetic testing is required to confirm the diagnosis (11, 12).

Since its initial description in the 1970s, only a handful of cases have been reported worldwide (10, 12–16). Here, we describe a family carrying a previously unreported missense substitution located close to an exon–intron boundary of the *CEBPE* gene at 14q11.2 (p.K170N; c.510G > C) and predicted to weaken a splice donor site. Among four siblings, two exhibited the characteristic clinical manifestations of the disease, consisting of recurrent pyogenic skin and visceral infections.

## Case report

A 37-year-old woman presented to the dermatology department with disseminated cutaneous lesions at various stages of evolution, including pustules, ulcerated and draining abscess-forming cysts, as well as mutilating atrophic scars involving the forearms, abdomen, trunk, and the medial and dorsal aspects of the thighs (Figure 1). She was referred to our department after repeated positive bacteriological cultures for *Staphylococcus aureus*, including a methicillin-resistant strain identified on the most recent test.

**Figure 1:**
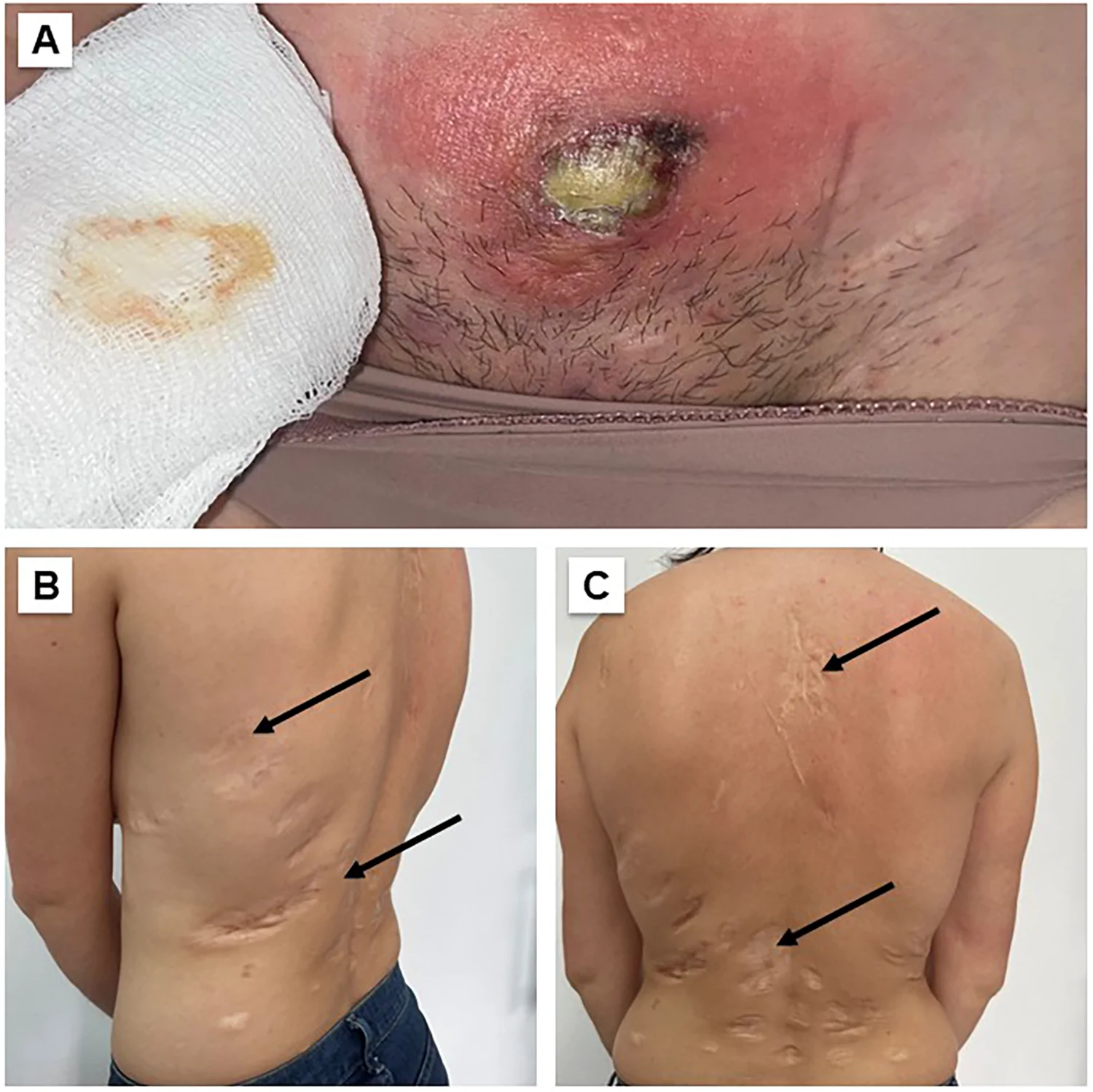
Clinical presentation of the female patient. **(A)** Inguinal abscess at presentation. **(B)** and **(C)** Atrophic cicatricial plaques consistent with abnormal wound healing following recurrent pyogenic soft-tissue infections.

The patient reported a lifelong history of large, persistent, and recurrent pyogenic cutaneous infections, first manifesting at the age of three years. Over time, she required multiple courses of antibiotic therapy and repeated surgical drainage procedures. Lesions were typically precipitated by minor trauma, such as shaving or small cuts.

Family history was notable, as one of her four siblings had also experienced recurrent pyogenic infections (Figure 2). The patient's brother was examined and exhibited a similar clinical pattern, with a history of recurrent skin infections complicated by osteomyelitis and pulmonary abscesses, as well as osseous deformities of the forearm secondary to osteomyelitis, resulting from contiguous spread of cutaneous infections (Figure 3). Since disease onset at the age of three years, he had also received multiple courses of antibiotic therapy and had been assigned various provisional diagnoses over the years.

**Figure 2:**
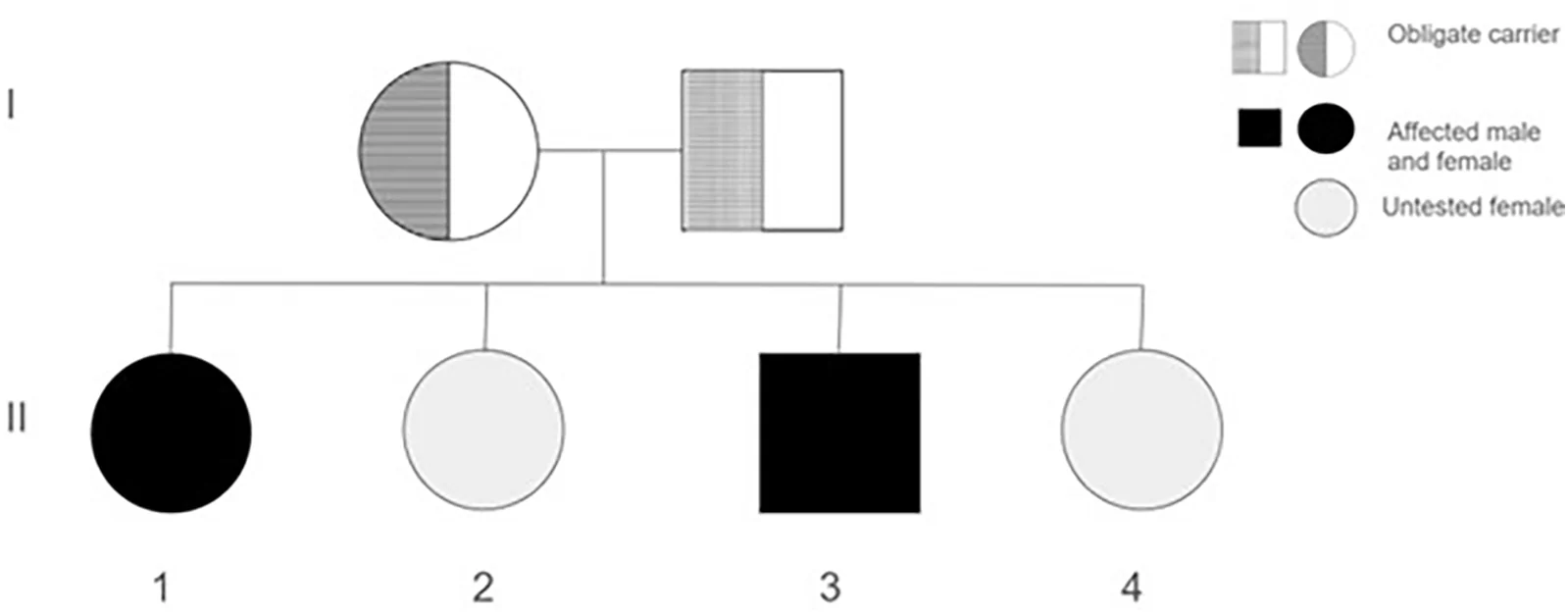
Family pedigree

**Figure 3:**
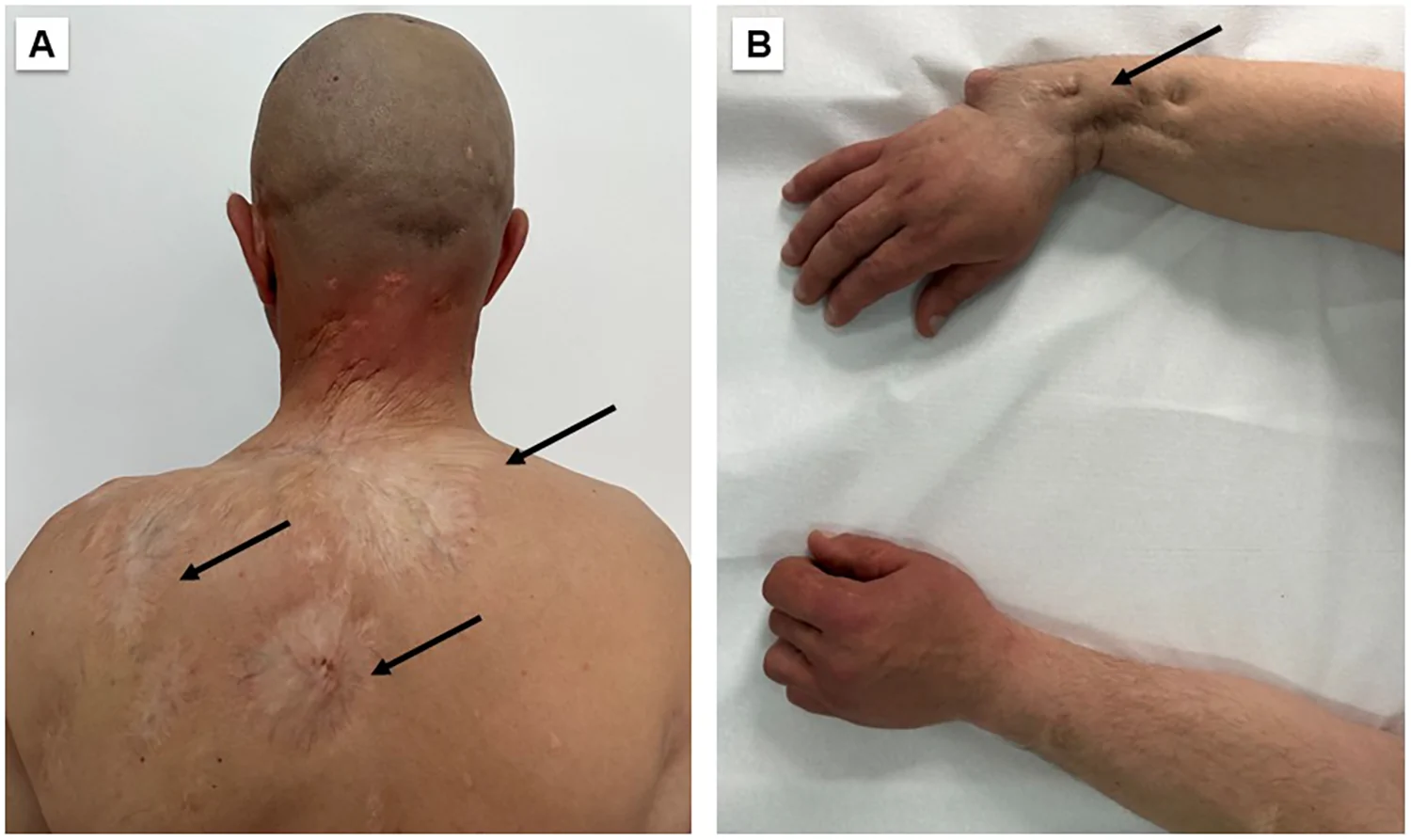
Clinical presentation of the male patient. **(A)** Altropic cicatricial plaques consistent with abnormal wound healing following recurrent pyogenic soft tissue infections. **(B)**Right radial deformity secondary to osteomyelitis resulting from contiguous spread of a soft-tissue infection.

The differential diagnosis included chronic inflammatory dermatoses prone to secondary infection, such as hidradenitis suppurativa and severe follicular occlusion disorders, as well as acquired causes of recurrent cutaneous infections, autoinflammatory syndromes, and neutrophilic dermatoses. However, the early onset in childhood, the lifelong history of recurrent skin infections, poor response to conventional therapies, and a suggestive familial pattern strongly suggested an underlying primary immunodeficiency, including conditions such as chronic granulomatous disease or hyper-IgE syndromes. Therefore, both patients were referred to the hematology department for further evaluation.

The laboratory investigations showed mild neutropenia (absolute meutrophil count1520/uL, 30% of differential leukocyte count). The serum immunoglobulin levels were within the normal range. The peripheral blood smear revealed hypogranular neutrophils with bilobed nuclei (Figures 4A–D), while immunophenotypically the neutrophil population demonstrated aberrant CD14 and diminished CD15 expression (Figure 4E). No aditional immunophentypical abnormalities were detected in the lymphocyte populations. Neutrophil oxidative burst testing could not be performed, as this assay is not currently available in our country. Whole exome sequencing was performed using the Blueprint Genetics Whole Exome Plus assay and identified a previously unreported missense substitution in the splice region of the *CEBPE* gene at 14q11.2 (p.K170N; c.510G > C) (Figure 5). At the time of the initial testing, the variant was absent from gnomAD and was initially classified as a variant of uncertain significance (VUS). Following confirmation of the same homozygous variant in the affected brother, the variant was reclassified as likely pathogenic according to the 2015 ACMG guidelines, based on criteria PM2, PP1, PP2, PP3 and PP4.

**Figure 4:**
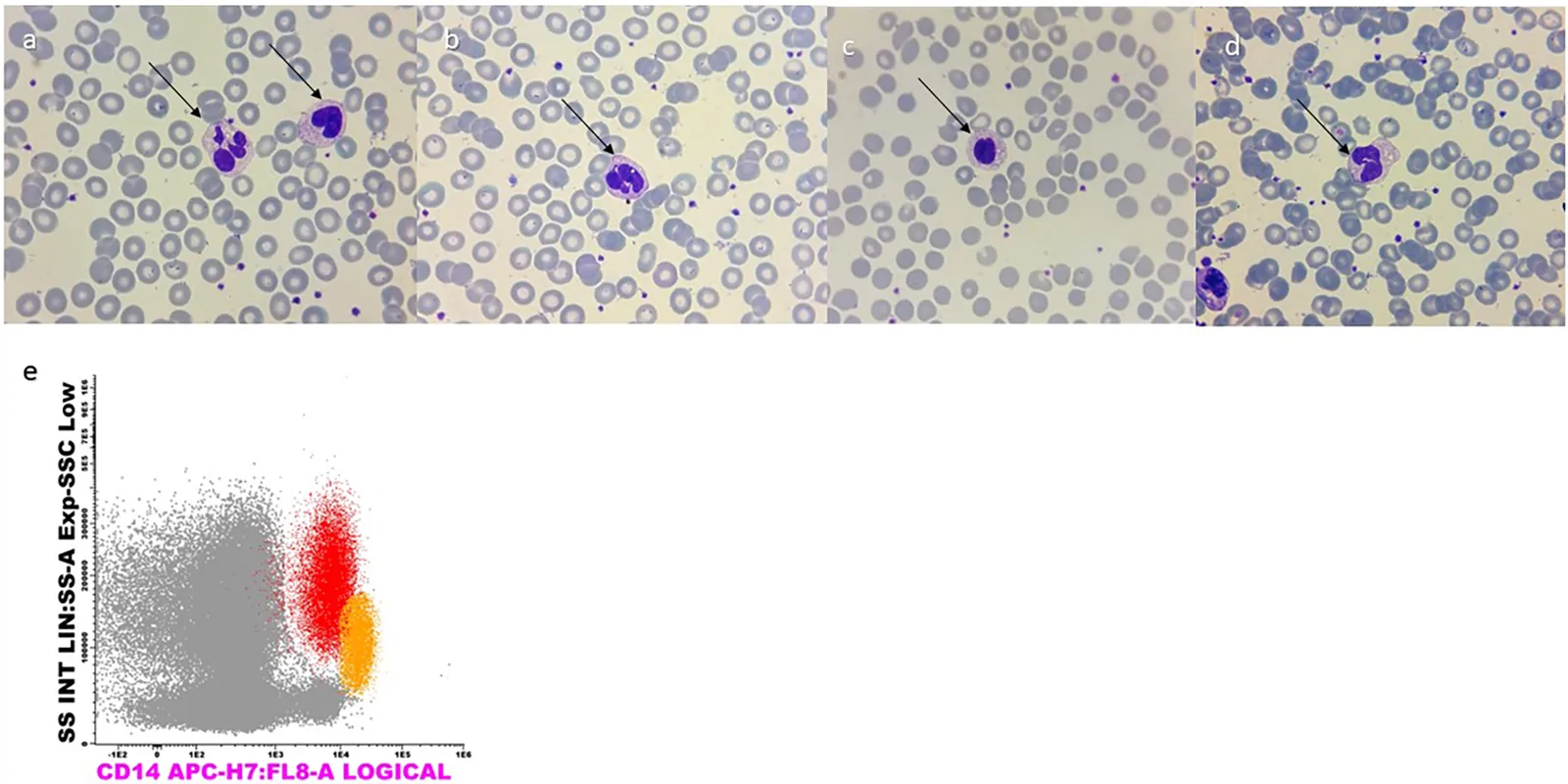
a–d Peripheral blood smear showing hypogranular neutrophils with abnormal nuclear segmentation, including bilobed nuclei (May–Grünwald–Giemsa stain; ×1,000 magnification, oil immersion). e. Flow cytometry plot demonstrating aberrantly bright CD14 expression in the neutrophil population (red) compared with monocytes (orange).

**Figure 5:**
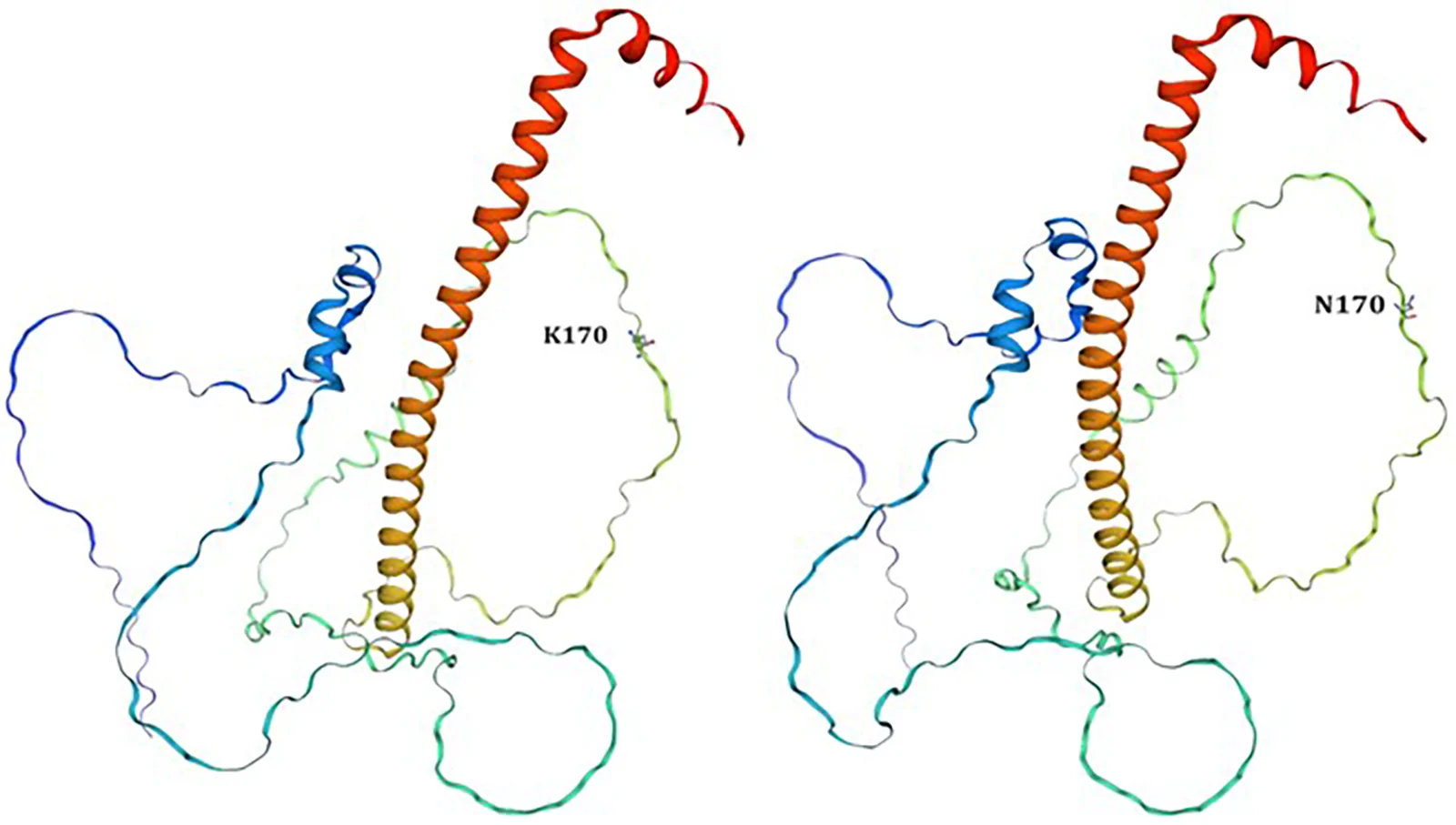
Conformatio changes between wild type *CEBPE* protein (left), versus mutant *CEBPE* protein induced by missense mutation in the splice region at 14q11.2 (p.K170N;c.510G>C). Generated with the automated protein structure homology-modeling server Swiss-Model.

Considering the findings, a diagnosis of neutrophil-specific granule deficiency was established. Management included the use of antibacterial cleansers, prophylactic antibiotic therapy, strict avoidance of shaving, and prompt initiation of antibiotics at the first signs of infection.

## Discussion

Immunodeficiency syndromes comprise a heterogeneous group of disorders characterized by impaired development or function of one or more components of the immune system, leading to increased susceptibility to infections, autoimmunity, and malignancy (17). These conditions are broadly categorized as primary (inborn errors of immunity), resulting from germline mutations that disrupt immune pathways, and secondary immunodeficiencies, which are acquired due to external factors such as infections, malignancy, or immunosuppressive therapy (2, 17).The occurrence of recurrent infections with onset in early childhood, particularly in the context of a suggestive family history, should prompt consideration of an underlying inborn error of immunity. Neutrophils are key effector cells of the innate immune system, essential for host defense against pathogens and for facilitating tissue repair. The cytoplasmic granules of neutrophils are critical to maintaining their normal morphology and mediating their antimicrobial functions (18, 19). During granulopoiesis, cytoplasmic primary granules first appear in promyelocytes. These primary, azurophilic granules are rich in myeloperoxidase and other antimicrobial enzymes.As promyelocytes mature into myelocytes, secondary or specific granules are formed, characterized by the presence of lactoferrin. Additional tertiary granules form in later maturation stages (20). The CCAAT/enhancer-binding protein (C/EBP) family plays an essential role in the terminal differentiation and functional maturation of neutrophils, including granule formation (8). Consequently, loss-of-function mutations in the *CEBPE* gene lead to the development of specific granule deficiency (3, 4, 7, 21–23).

So far fewer than 10 families with pathogenic *CEBPE* variants causing specific granule deficiency have been described in the literature. Clinically, affected individuals typically manifest early-onset pyogenic infections affecting the skin, visceral organs, and occasionally the central nervous system.

The clinical and morphological phenotype observed in this family is consistent with classical CEBPE-associated SGD and resembles that reported with biallelic loss-of-function variants, including c.249_253del, c.509dup, c.742_747del and c.403C > T, which have been associated with recurrent pyogenic infections and characteristic abnormalities of neutrophil granulation and nuclear morphology (3, 4, 7, 21–23). The phenotypic spectrum of CEBPE-related disease nevertheless extends beyond classical SGD. The heterozygous p.(Val218Ala) variant has been associated with SGD characterized by abnormal nuclear lobulation and near-absence of secondary granules (24), whereas the homozygous gain-of-function p.(Arg219His) variant causes a distinct syndrome of autoinflammation, immunodeficiency and neutrophil dysfunction (23). Our findings therefore extend the genotypic spectrum of CEBPE-associated SGD by identifying the homozygous c.510G > C [p.(Lys170Asn)] variant in affected members of the same family.

In this case, the reliance on clinical features alone was insufficient to establish a definitive diagnosis, underscoring the necessity of comprehensive immunological and genetic investigations for mutation identification, as well as the pivotal role of coordinated multidisciplinary management involving dermatology, hematology, and infectious diseases.

An important limitation of this report is the absence of functional validation of the c.510G > C variant. Although the variant results in the p.(Lys170Asn) substitution is potentially resulting in aberrant splicing, transcriptional studies were not performed. Thus, neither the predicted splicing defect nor the functional consequences of the amino-acid substitution were experimentally demonstrated. The proposed pathogenicity of the variant should therefore be interpreted in the context of familial segregation, the marked concordance between genotype and phenotype, rather than direct mechanistic evidence.

## Data Availability

The original contributions presented in the study are included in the article/Supplementary Material, further inquiries can be directed to the corresponding authors.
